# Long-term preservation of biomedical research data

**DOI:** 10.12688/f1000research.16015.1

**Published:** 2018-08-29

**Authors:** Vivek Navale, Matthew McAuliffe

**Affiliations:** 1 Center for Information Technology, National Institutes of Health, Bethesda, Maryland, 20892, USA

**Keywords:** Open, Archival, Information, System, Biomedical, Data, Preservation, Access

## Abstract

Genomics and molecular imaging, along with clinical and translational research have transformed biomedical science into a data-intensive scientific endeavor. For researchers to benefit from Big Data sets, developing long-term biomedical digital data preservation strategy is very important. In this opinion article, we discuss specific actions that researchers and institutions can take to make research data a continued resource even after research projects have reached the end of their lifecycle. The actions involve utilizing an Open Archival Information System model comprised of six functional entities: Ingest, Access, Data Management, Archival Storage, Administration and Preservation Planning.

We believe that involvement of data stewards early in the digital data life-cycle management process can significantly contribute towards long term preservation of biomedical data. Developing data collection strategies consistent with institutional policies, and encouraging the use of common data elements in clinical research, patient registries and other human subject research can be advantageous for data sharing and integration purposes. Specifically, data stewards at the onset of research program should engage with established repositories and curators to develop data sustainability plans for research data. Placing equal importance on the requirements for initial activities (e.g., collection, processing, storage) with subsequent activities (data analysis, sharing) can improve data quality, provide traceability and support reproducibility. Preparing and tracking data provenance, using common data elements and biomedical ontologies are important for standardizing the data description, making the interpretation and reuse of data easier.

The Big Data biomedical community requires scalable platform that can support the diversity and complexity of data ingest modes (e.g. machine, software or human entry modes). Secure virtual workspaces to integrate and manipulate data, with shared software programs (e.g., bioinformatics tools), can facilitate the FAIR (Findable, Accessible, Interoperable and Reusable) use of data for near- and long-term research needs.

## Introduction

Over the past decade, major advancements in the speed and resolution of acquiring data has resulted in a new paradigm, ‘Big Data.’ The impact of Big Data can be seen in the biomedical field. Billions of DNA sequences and large amounts of data generated from electronic health records (EHRs) are produced each day. Continued improvements in technology will further lower the cost of acquiring data, and by 2025, the amount of genomics data alone will be astronomical in scale ^1^. In addition to large data sets and the large number of data sources, challenges arise from the diversity, complexity and multimodal nature of data generated by researchers, hospitals, and mobile devices around the world. Research programs like the All of Us Research Program envision using Big Data to transform healthcare from case-based studies to large-scale data-driven precision medicine endeavors ^2^.

Harnessing the power of digital data for science and society, requires developing management strategies that enable data to be accessible and reusable for immediate and future research needs. With the preponderance of bigger datasets, the volume, variety and magnitude of biomedical data generation is significantly higher than existing analytical capabilities. The time lag between data accumulation and thorough analysis will result in more data being passive or inactive for extended time intervals. Meaningful associations for data reuse for applications beyond the purpose for which it was collected will also be a time-intensive endeavor. Therefore, our opinion is that attention should be focused on developing a data preservation strategy that can ensure biomedical data availability for longer term access and reuse.

## Model for long-term data preservation

Challenges to manage vast amount of data from space missions led to the development of the Open Archival Information System (OAIS) model ^3^. The OAIS model defined “as an archive that consists of an organization of people and systems with responsibility to preserve information and make it available for a designated community” provides the framework for long term preservation of data ^4^.

**Figure 1.**
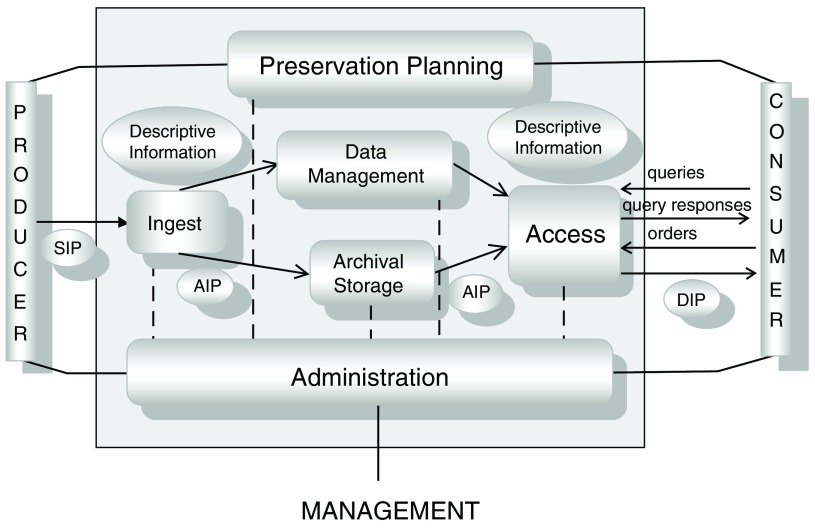
Open Archival Information System (OAIS) functional model. Information flow within the OAIS model is by means of “packages”, SIP, AIP and DIP with the related interfaces (both solid and dotted lines) that show the interaction between the various functions ^5^. Various OAIS implementations have led to development of digital repository systems (e.g. Dspace, Fedora) and customized repositories (e.g. the US National Oceanic and Atmospheric Association). Reproduced with permission from The Consultative committee for Space Data Systems ( https://public.ccsds.org/pubs/650x0m2.pdf). The source for this OAIS implementation was originally provided by Ball (2006) ( http://www.ukoln.ac.uk/projects/grand-challenge/papers/oaisBriefing.pdf) ^6^.

The functional model ( Figure 1) illustrates that during the Ingest process, Submission Information Packages (SIP) are produced. Metadata and descriptive information are important for developing Archival Information Packages (AIP) for data storage. Metadata can include attributes that establish data provenance, authenticity, accuracy, and access rights. The Dissemination Information Packages (DIP) are produced in response to queries from consumers. The OAIS model includes six functions (shown in Figure 1) - Ingest, Access, Data Management, Archival Storage, Administration and Preservation Planning. Figure 1 contains a pictorial representation of the OAIS model.

The wide variety of examples illustrate that the OAIS model is content and technology agnostic. Therefore, we posit that the model can be used for developing biomedical digital data preservation strategy. In the following sections, we contextualize the functional aspects of the model needed for successful implementation of biomedical data repository ecosystems.

## Preservation planning

As shown in Figure 1, preservation planning is an important bridge between the data producers and consumers. During the planning stage several questions (some of which are listed below) must be addressed:

• How will data be collected and managed?
• What data (and metadata) is required for establishing provenance?
• What type of common data elements and bio-ontology are needed?
• How will data curation be carried out for the data sets?
• Which data types will be stored and preserved?
• How will data access be provided?
• What methods are needed to maintain data quality?

In the past these questions have been the responsibility of biomedical data custodians and curators working in libraries, archives and repositories, who are usually engaged during the latter part of data lifecycle management (during data preservation and access services). We think that importance should be placed on data preservation during the planning of initial activities (e.g. collection, processing, storage), along with the ensuing activities (data analysis, sharing and reuse). In our opinion, developing a community of data stewards for biomedical research programs within institutions is an important step towards long-term preservation of biomedical data.

Considering the interdisciplinary skill set needed for data stewards, we propose that institutions leverage the expertise of staff (e.g. biologists, physicians, informaticians, technologists, library science specialists, etc.) for their respective biomedical research programs. We envision data steward teams to be engaged early in research data lifecycle management, developing digital data stewardship plan(s) for biomedical data sets. These activities can promote a culture of semantic scientists for biomedical programs, which can help reduce the time and cost of data interpretation by biocurators ^7^.

We think that establishing data stewards’ responsibility within the biomedical research program can improve data quality, provide traceability, and support reproducibility.

Typically, a designated community for biomedical data are researchers of a sub-discipline for a disease (e.g. cancer). Reviewing the research sponsors’ requirements, understanding the volume and types of data to be collected, and defining how the data will be organized and managed can all promote the reuse of data ^8^.

Goodman *et al*. provide a short guide to consider when caring for scientific data. The guide highlights the use of permanent identifiers, depositing data in established repository archives and publishing code and workflows that can facilitate data use/reuse ^9^.

It is also essential that research group leaders and institutions emphasize data management best practice principles ^10^. An important practice for ensuring good research management in laboratories includes selecting the right medium (paper-based and/or electronic) for laboratory notebooks ^11^.

## Administration

Both producers and consumers of data will be best served by implementing established procedures for digital preservation. Producers of biomedical data should develop a comprehensive data management plan (DMP) that addresses policies and practices needed to acquire, control, protect and deliver data, and the steps needed for the preservation and reuse of data ^12^.

As a first step, data stewards should establish a DMP to identify the types of data that will be collected, provide information on the organization of data, assign roles and responsibilities for description of the data and document processes and procedures for Ingest and methods for data preservation and dissemination.

Data collection strategies need to be established in context of institutional policies for biomedical archives. We recommend that DMP be used as a planning tool to communicate all operations performed on data, and details of software used to manage data. Williams *et al*. provide a comprehensive review of data management plans, their use in various fields of biomedical research, and reference material for data managers ^13^.

As part of administration, data stewards should engage with a designated community (data creators, funding agencies, stakeholders, records managers, archivists, information technology specialists) to appraise the data to determine whether all the data produced during the research program should be preserved, or whether different data types (raw, processed, etc.) require different degrees of preservation (e.g. temporary with a time stamp for review or permanent indefinitely).

Establishing data provenance should be part of data collection and management strategy. This may not be always easy, because contextual information (metadata) about experimental data (wet/dry lab) and workflows is often captured informally in multiple locations, and details of the experimental process are not extensively discussed in publications. Contacting the original source for additional information may or may not yield fruitful results, and the reproducibility of experiments becomes challenging in many cases.

Security controls should be part of data collection and management strategy. For initial security controls assessment, guidance documents, FISMA, NIST-800-53 and FIPS, can provide tools for an organizational risk assessment and validation purposes ^14, 15^. A wide range of issues involving ethical, legal and technical boundaries influence biomedical data privacy, which can be specialized for the type of data being processed and supported ^16^. Important points to consider are confidentiality, disclosure specifications, data rights ownership, and eligibility criteria to deposit data to an established repository.

## Ingest

Capturing relevant data from the experiment in real time can be one of the better practices for establishing biomedical data provenance. Automated metadata capture when possible (using a laboratory information management system), and digitization where automation is not possible, can reduce errors, minimize additional work and ensure data and metadata integrity ^17^. We believe that establishing data provenance will result in successful preparation of SIP during ingest ( Figure 1).

SIP for clinical research, patient registries and other human subject research can be developed by use of common data elements (CDEs). A CDE is defined as a fixed representation of a variable to be collected within a clinical domain. It consists of a precisely defined question with a specified format or a set of permissible values for responses that can be interpreted unambiguously in human and machine-computable terms. There are many examples of CDE usage and information on CDE collections, repositories, tools and resources available from the National Institutes of Health (NIH) CDE Resource Portal ^18^. The advantage of using CDEs was highlighted by the Global Rare Disease Repository (GRDR), where researchers integrated de-identified patient clinical data from rare disease registries and other data sources to conduct biomedical studies and clinical trials within and across diseases ^19^.

Ontologies are useful for annotating and standardizing the data description so that the querying and interpretation of data can be facilitated. Selecting a biontology requires knowledge about the specific domain, including current understanding of biological systems. Several ontologies have been reported for various biological data and can be selected for research data ^20^. An online collaborative tool (e.g. OntoBrowser) can be used to map reported terms to preferred ontology (code list), which can be useful for data integration purposes ^21^. We believe that use of CDEs and Ontologies can result in developing AIP for long-term preservation of biomedical data.

## Data management

Data authenticity, accuracy and reliability influence data quality. For that purpose, controls from the very beginning of research (as part of DMP) need to be established. For experimental work, instrument calibration and validation of data analysis methods contributes significantly to the quality of data produced in a lab. Currently, many approaches for data quality assessment exist and their strengths and weakness have been discussed ^22^. The most common approach for obtaining a first look at the quality of new data is by reviewing supporting data provided with research articles that contextualizes data to support the research goals and conclusions. Additional quality assessment is obtained by the evaluation provided by data producers and data curators and, when appropriate, with automated processes and algorithms.

In the context of Electronic Health Records (EHR), the five dimensions of data quality are: completeness, correctness, concordance, plausibility and currency. The data quality assessment of these dimensions has been carried out by one or more of the seven categories: comparison with gold standards, data element agreement, data source agreement, distribution comparison, validity checks, log review, and element presence ^23^. Validated and systematic methods for EHR data assessment are important, and with shared best practices, the reuse of EHR data for clinical research can be promoted.

We think data curation needs should be assessed during DMP. One of the ways to assess data curation is by using a Data Curation Maturity model ^24^. The model assumes that new areas of research (evolving areas) may not have best practice(s) from the very beginning, but having an indicator to show maturity levels at different stages of an organization or group in performing tasks enables in improving curation practices. A staging approach is proposed to aid in developing good practices (and even best practices) and identify ineffective practices for various tasks so that the quality of data can be improved upon from the beginning. The maturity model can be useful for determining steps that are needed to improve data quality.

We opine that data stewards should engage with established repositories and develop data sustainability plans. Many well-known biomedical repositories are known to host wide ranges of biomedical data (for example, GenBank for nucleotide sequence, Gene Expression Omnibus for microarray and high throughput gene expression data, miRbase for annotated sequences, dbSNP for single nucleotide polymorphism (SNPs), Protein Data Bank for 3D structure data for macromolecules (proteins and nucleic acids), RefSeq for non-redundant DNA, RNA and protein sequences. Additionally, disease-specific repositories for traumatic brain injury and Parkinson’s disease are also available ^25^.

A tabulated listing of 21 established life science repositories with various types of user support services (e.g. for visualization, data search, analysis, deposition, downloads, and online help) is also available ^26^. Additional helpful resources, re3data.org: the Registry of Research Data Repositories, can be used to identify appropriate repositories for storage and search of research data ^27^.

## Archival storage

Both raw and processed data are produced during biomedical research. Therefore, developing a storage roadmap is important and should consider data types, volume, data format and the applications required for current and future processing. Broadly, file, object and block are three types of data storage options available to biomedical researchers ^28^.

File Storage has been used for storing large and smaller scale biomedical datasets providing direct and rapid access to local computing resources, including High Performance Computing clusters. Object Storage is ideal for systems that need to grow and scale for capacity. Block Storage is useful when the software application needs to tightly control the structure of the data, usually the case with databases.

Depending on access needs a tiered data storage strategy can be used for migrating data from high input/output (I/O) disks to lower I/O media, like magnetic tapes. Data Storage strategy should consider at least two types of media (disks and tapes) to mitigate the probability of data loss due to media failure. In addition, primary and backup copy of data should be stored at two different geographically separated locations (at least several hundred miles apart).

**Figure 2.**
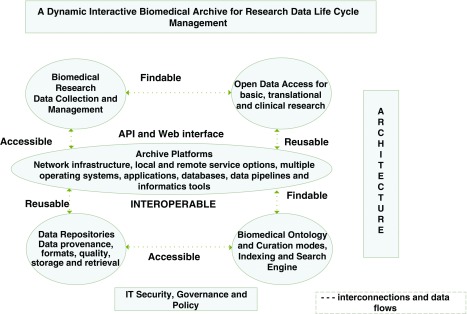
Conceptual model of bio-archive platform powered by cloud resources for long-term preservation of biomedical needs. Figure adapted from Navale and Bourne (2018) ^30^.

Considering the diversity, complexity and increasing volume of biomedical research data, we posit that cloud based platforms can be leveraged to support varieties of ingest modes (e.g. machine, software or human entry modes) to make data findable, accessible, interoperable and reusable (FAIR) ^29^. In our opinion, a cloud-based data archive platform (shown in Figure 2) can provide a dynamic environment for managing research data life cycle along with capabilities for long-term preservation of biomedical data ^30^.

## Access

Needs in biomedical research can vary from simple queries (as shown in Figure 1) to a wide range of capabilities (workflow and software tools) usually employed for analysis of large scale data sets (genomics data) ^31^. Dissemination information package (DIP) can result from discovery search engines, e.g. DataMed ^32^, and machine readable methods (e.g. repositive.io) for extracting new knowledge from the datasets ^33^, with online available resources for digital sharing purposes ^34^.

Broadly speaking, access to data and metadata can be discussed in terms of the web and Application Programming Interface (API). In the web mode ( Figure 2), user utilizes an interactive “browser” that presents overviews, summaries, and familiar search capabilities. In the API mode, the same underlying data and metadata can be consumed by a computer. The API mode is composed of a set of protocols and instructions that can serve the needs of both software developers and users. APIs commonly use Representational State Transfer (REST) ^35^. REST utilizes the standard ‘http’ protocol access to manipulate data or metadata, and standards and toolsets for developing, documenting, and maintaining REST-based APIs are available ^36^. In our opinion, type of API adoption will be driven by research questions and user community needs, evident from the comparison of three Genomics API’s (Google Genomics, SMART Genomics, and 23andMe) ^37, 38^.

For longer term access needs using file formats that have a good chance of being understood in the future is one of the ways of overcoming technology obsolescence. File formats that have characteristics of “openness”, “portability”, and maintain “quality” are better choices for long term preservation needs.

Information on the data types (e.g. text, image, video, audio, numerical), structure and format are essential for ensuring that it can be used and reused over time. Access to data will be greatly enhanced if data are archived in “open formats” not restricted by proprietary software, hardware, and/or by purchase of a commercial license. Some examples of open data formats in use are: comma or tab-separated values (csv or tsv) for tabular data, hierarchical data format and NetCDF for structured scientific data, portable network graphics for images, Open Geospatial Consortium format for geospatial data, and extensible markup language for documents ^39^. If proprietary formats are used for initial data collection and analysis work, it should be exported to an open format for archival purposes. In some cases, proprietary formats have become standard formats when popularity and utility have driven tools and algorithms purpose-built to ingest and modify those formats (e.g., Affymetrix .CEL and .CDF formats).

We also think that the reuse of preserved data can be enhanced by the open availability of client software to user communities. One example is Bioconductor for genomic data ^40^. In addition, developing and applying ontology-driven transformation and integration processes can result in open biomedical repositories in semantic web formats ^41^.

## Conclusion

Valuing, protecting, enabling access, and preserving data resources for current and future needs of researchers, laboratories, institutes and citizens is a critical step in maturing the biomedical research process of any organization or community.

With advent of Big Data, biomedical researchers need to become more proficient in understanding and managing research data throughout its lifecycle. Establishing the responsibilities of data stewards within the biomedical research program can improve data quality, provide traceability and support reproducibility. Determining specifically what to preserve and for how long are policy decisions that require data steward teams to engage with funding agencies, designated communities and established repositories.

We opine that the likelihood of maintaining the authenticity, accuracy and reliability of biomedical data for longer-term access will be enhanced by application of the OAIS model. Implementation of the model for biomedical data sets will provide renewed opportunities for data integration, analysis and discovery for basic, translational and clinical research domains.

## Data availability

No data are associated with this article.
