# Supplementary material for: Long-term preservation of biomedical research data
Source: F1000Res. 2018 Aug 29;7:1353. [Version 1] doi: 10.12688/f1000research.16015.1 (PMC6144948; doi:10.12688/f1000research.16015.1)
Supplement: Peer review history

# Peer review: Referee response for version 1

**DOI:** 10.5256/f1000research.17492.r38251

**Published:** 2018-10-09

**Authors:** Ravi Madduri

**Affiliations:** 1 Computation Institute, University of Chicago, Chicago, IL, USA

The article titled "Long-term preservation of biomedical research data” by Navale et.al, is a timely article that highlights the need for a long term strategy for preservation of data products generated from research projects. Often times a lot of time is spent in the initial activities of a research project which involves data collection, processing, analysis, sharing and publishing but substantially less time is spent in curating the data, making data reusable and finally long term preservation of data products. The paper presents a strategy for long term data preservation which the authors have broken down into multiple stages. This reviewer agrees with the strategy and the overall presentation of the strategy in the manuscript. There is, however, one important challenge in long term data management that this reviewer felt has not been covered adequately which is the economics of long term data preservation. Determining which data products are preserved and for how long is an important part of the puzzle. Additionally, the economics of long term storage along with who the stakeholders are and what the incentives for this to happen is also important. The article would be made better with these additions.

I have read this submission. I believe that I have an appropriate level of expertise to confirm that it is of an acceptable scientific standard.

# Peer review: Referee response for version 1

**DOI:** 10.5256/f1000research.17492.r37766

**Published:** 2018-09-24

**Authors:** David Giaretta

**Affiliations:** 1 Primary Trustworthy Digital Repository Authorisation Body Ltd (PTAB Ltd), Dorset, UK

The reason I ticked "Partly" in the above checkboxes is that the article has omitted clear discussion of the key concepts in OAIS for preservation, which are also key to the "R" in FAIR i.e. Reuse.

 These may be addressed by including a discussion of the OAIS Information Model. 

 OAIS defined Long Term Preservation as *The act of maintaining information, Independently Understandable by a Designated Community, and with evidence to support its Authenticity, over the Long Term.*

 To explain what I mean briefly, to ensure understandability the archive should collect the appropriate Representation Information, and ensure that as the  Designated Communities Knowledge Bases, the Representation Information must be supplemented. For example if the ontology, which is used to understand the biomedical data, goes out of use over time, for example the URL for its location no longer works, the archive will need to ensure that the original ontology remains available. 

 Similarly, as evidence for Authenticity the archive should collect Provenance about the data, as is briefly mentioned in the article.

 The Archival Information Package, which is the AIP shown in the Functional Model, is a way to ensure that the archive has captured all the information required for Long Term Preservation, including Representation Information, Provenance Information and several other items. In order to create the AIP for a dataset the archive should ensure that the required information is captured during Ingest, and is maintained over time.

 The data management plan should help to ensure that the appropriate information is captured over the course of the project in order to provide to the archive.

 One last point which is worth mentioning is that the evaluation of the ability of an archive is also possible through ISO 16363 audit and certification.

I have read this submission. I believe that I have an appropriate level of expertise to confirm that it is of an acceptable scientific standard, however I have significant reservations, as outlined above.

## References

[1] Standard ISO: 14721: International Organization for Standardization: 2012: Space Data and Information Transfer Systems - Open Archival Information System Reference Model.2012;

# Peer review: Referee response for version 1

**DOI:** 10.5256/f1000research.17492.r37763

**Published:** 2018-09-19

**Authors:** Chaitan Baru

**Affiliations:** 1 San Diego Supercomputer Center, University of CaliforniaSan Diego, La Jolla, CA, USA

In sum, this paper is highlighting the importance and need for "data stewardship", viz., well-considered data management plans for every project that produces/generates data. Data stewardship plans should be developed early in a project cycle--indeed, along with the development of the science/research goals of the project itself.

 The authors caution that this problem becomes even more urgent in the era of "big data". They recommend use of an existing approach, viz., the Open Archival Information System (OAIS).

 The issues mentioned and the approaches suggested are very reasonable, and very much in step with similar concerns and approaches in several other domains, which are all facing the data deluge.

 In fact, I have heard so much discussion and read so many articles on this topic--supporting the approaches described in this paper as well--that I am now concerned that, as a community, we are probably not taking the right approach to this problem.

 First, the only way that the community may pay attention and spend resources on this problem is if they see value. These type of articles should probably begin with the value of doing this work, rather than the cost. Almost all articles on this topic talk in detail about the costs, and simply presume that the value exists. Value could be demonstrated by showing real science examples that benefited from archival data; examples of studies that went into archival data and found something new and interesting. Or, conversely, studies that duplicated effort, or failed in other ways, for not digging into archival data.

 Second, curation is not a static process. The costs of curation, done properly, may actually be way more than what these papers suggest. Since science is not static, the relevance or "meaning" of a particular dataset is also not static. Data may become less or more valuable as the field progresses. All of that speaks to what I would call "continuous curation" of scientific data, and not just one-time curation at the time of creation.

 Finally, what to do when everyone is extolling the need and virtue of curation but no one is spending nearly enough  resources to do the job right. One would think that this is the classic use case for AI and "smart" techniques. Why not let the computer do the job that no human is willing or able to do for the amount of money we are willing to spend. Rather than AI, this may be the classic use case for IA--intelligence augmentation, with a human in the loop.

I have read this submission. I believe that I have an appropriate level of expertise to confirm that it is of an acceptable scientific standard.

# Peer review: Referee response for version 1

**DOI:** 10.5256/f1000research.17492.r38250

**Published:** 2018-09-17

**Authors:** George Alter

**Affiliations:** 1 Inter-university Consortium for Political and Social Research (ICPSR), University of Michigan, Ann Arbor, MI, USA

This article explaines the importance of engaging data stewards in planning for data preservation at the beginning of data collection. It makes an important contribution by offering useful guidance about each stage of the data life cycle, such as the use of CDEs and ontologies, the Data Curation Maturity model, and open formats. Data would be more plentiful, better documented, and easier to reuse if this planning took place.  

 The following suggestions are intended to strengthen and expand the current draft of the paper.  Since this is explicitly an "opinion article," some of these comments reflect my own opinions, which the authors may not share.

 1. Under "Mandatory Responsibilities" the OAIS standard says that an archive must: "Ensure that the information to be preserved is Independently Understandable to the Designated Community. In other words, the community should be able to understand the information without needing the assistance of the experts who produced the information." I think a statement like this provides a focus for the activities involved in preparing data for prservation. OAIS is not simply about assuring that the data survive. Preservation also assures that the data will be reusable (the 'R' in FAIR) in the future. It might be worthwhile to point out the relevance of OAIS for FAIR earlier in the paper.

 2. Although Figure 1 suggests that the SIP arrives at the archive fully formed, the text of the OAIS standard emphasizes that the relationship between the archive and the data producer may involves a lot of negotiation. Data repositories often need to contact the data producer several times to get the information that they need. This is a costly process, and the recommendations in this paper would reduce those costs. This is worth mentioning, because data producers often only see the costs of preparing data for sharing.

 3. The discussion of CDEs and ontologies could also mention the Center for Expanded Data Annotation and Retrieval (CEDAR), which has developed tools for creating metadata.

 4. When choosing a data repository, data stewards should favor repositories that offer an assurance of permanence and trustworthiness. This is especially important in the biomedical community, because valuable data have been lost when repositories and databases closed. There are several bodies that certify data repositories as trustworthy. ISO has a "Standard for Trusted Digital Repositories" (ISO 16363), which is very comprehensive and usually involves an external auditor. The CoreTrustSeal has a smaller list of requirements and relies on a self-audit.

 5. Confidentiality and disclosure are mentioned in a paragraph about security controls, but this deserves a little more space. Data producers set the terms of future reuse of data when they make informed consent agreements with subjects. If data sharing is not anticipated in the informed consent agreement, it is very difficult to share the data. Since the informed consent agreement is supervised by an IRB, the IRB should also approve plans for future data sharing. These plans could involve an array of legal (data use agreements) and technical (anonymization, "data enclaves") measures to protect confidential information.

I have read this submission. I believe that I have an appropriate level of expertise to confirm that it is of an acceptable scientific standard.

# Peer review: Referee response for version 1

**DOI:** 10.5256/f1000research.17492.r37761

**Published:** 2018-09-13

**Authors:** Jane Greenberg

**Affiliations:** 1 Metadata Research Center, College of Computing & Informatics, Drexel University, Philadelphia, PA, USA

This is a timely scientific undertaking and stands as an important, original contribution to the body of research on data preservation in the context of big data, biomedical research, and data management/archiving. Furthermore, the research makes important links to key underpinnings and research covering data quality, modeling, along with metadata, and ontologies.

 Much of the big data research to date has focused on algorithmic work, visualization, and related topics, whereas data preservation and archival research has been driven largely from the context of institutional repositories that are not necessarily storing big data.

 This paper bridges these two research areas, using the OAIS model as a platform to weave together these topics, and provide salient discussion about keys pillars of covering preservation planning, administration, ingest, data management, archival storage, and access. The research is further contextualized by the FAIR principles that seek to ensure that data not only findable and accessible, but also interpretable and reusable. Further, the authors hone in on the role of data modeling, metadata, including the provenance model and ontologies.

 The writing is excellent, the synthesis of the literature and integration of other research is solid. Additionally, the diagrams are illustrative. (Note, this my first review in this system, and I am super excited and pleased to have had the opportunity to serve as a reviewer for this original research, and eager to share with colleagues.  In fact, have already shared this link with colleagues, so they can include this piece as a key reading across related courses as our academic quarter at Drexel is almost underway).

I have read this submission. I believe that I have an appropriate level of expertise to confirm that it is of an acceptable scientific standard.
